# The Modification of Useful Injection-Molded Parts’ Properties Induced Using High-Energy Radiation

**DOI:** 10.3390/polym16040450

**Published:** 2024-02-06

**Authors:** Martin Bednarik, Vladimir Pata, Martin Ovsik, Ales Mizera, Jakub Husar, Miroslav Manas, Jan Hanzlik, Michaela Karhankova

**Affiliations:** 1 Faculty of Technology, Tomas Bata University in Zlin, Vavreckova 5669, 760 01 Zlin, Czech Republic; pata@utb.cz (V.P.); ovsik@utb.cz (M.O.); j_hanzlik@utb.cz (J.H.); 2 Faculty of Applied Informatics, Tomas Bata University in Zlin, Nad Stranemi 4511, 760 05 Zlin, Czech Republic; mizera@utb.cz (A.M.); husar@utb.cz (J.H.); manas@utb.cz (M.M.); m_karhankova@utb.cz (M.K.)

**Keywords:** polymers, beta radiation, injection molding, cross-linking, oxidation, regression, mechanical properties, surface properties

## Abstract

The modification of polymer materials’ useful properties can be applicable in many industrial areas due to the ability to make commodity and technical plastics (plastics that offer many benefits, such as processability, by injection molding) useful in more demanding applications. In the case of injection-molded parts, one of the most suitable methods for modification appears to be high-energy irradiation, which is currently used primarily for the modification of mechanical and thermal properties. However, well-chosen doses can effectively modify the properties of the surface layer as well. The purpose of this study is to provide a complex description of high-energy radiation’s (β radiation) influence on the useful properties of injection-molded parts made from common polymers. The results indicate that β radiation initiates the cross-linking process in material and leads to improved mechanical properties. Besides the cross-linking process, the material also experiences oxidation, which influences the properties of the surface layer. Based on the measured results, the main outputs of this study are appropriately designed regression models that determine the optimal dose of radiation.

## 1. Introduction

The ever-increasing consumption of polymer materials in the entire engineering industry continually places pressure on the rising requirements put on base materials. One of the options which can meet high technical requirements is the use of special, although expensive, and difficult-to-process polymer materials [1,2,3]. Another alternative could be the use of cheaper, commodity, and technical plastics in combination with a suitable type of modification, which can enhance the material throughout its volume and improve other mechanical and thermal properties [3,4]. These specific applications often require the joining of individual parts to larger assemblies. One of the most important methods for this kind of application is bonding. Unlike mechanical methods of connecting (welding, riveting), bonding introduces no additional tension, dampens vibrations, increases rigidity and buckling strength, and can be used for water- and gas-tight applications. Furthermore, mechanical joints come with the drawback of being heavier [5]. The bonding of commodity and technical plastics is usually preceded by suitable modifications [6], which target the material’s surface layer properties, such as the wettability of joined surfaces, and increase its free surface energy and adhesive properties [7].

If an application requires commodity and technical plastics, which can be polyethylene (PE), polyamide (PA), etc., to satisfy the aforementioned criteria with regard to the mechanical properties and quality of adhesive bonds, it is necessary to choose a suitable type of modification for both the mechanical and surface properties [3,4,8]. Current studies describe a wide spectrum of methods that primarily focuses on either the modification of mechanical and thermal properties [9,10,11] or on the alteration of surface properties [12,13,14]. Due to these modifications, many common polymers find their use not only in numerous engineering applications but also outside of these, for example, in the biomedical field [15] or energy industry [16].

Regarding the modification of a polymer’s surface, the most widely used methods are plasma treatment [8,13,15,17], corona treatment [12,14], or chemical etching [12]. The aforementioned methods are quite effective in the modification of surface layer properties; however, their potential in the adjustment of mechanical properties is significantly limited. It would be beneficial for industrial practice if one method could modify both the mechanical [18,19] and surface layer properties of injection-molded materials [13,14,17]. From this point of view, irradiation appears to be the most suitable method, as some recent studies suggest that its use with a correctly chosen radiation dose could lead to an improvement of not only the mechanical properties [18,19] but also lead to an effective modification of surface layer properties, such as wettability or free surface energy [5].

The process of radiation cross-linking of injection-molded parts manufactured from polymer materials is performed, exposing them to radiation (most commonly the electron beams from electron accelerators). As mentioned by Makuuchi et al. [20] and other authors [21,22], the primary interactions of accelerated electrons with polymer material are the ionization, excitation, stabilization, neutralization, and generation of free radicals. Free radicals can be created either due to the scission of a main chain or due to the dissociation of a side chain. Following the primary reactions are secondary reactions, mainly hydrogen abstraction, recombination (cross-linking or branching), chain scission, oxidation, and grafting. All of these processes and reactions evoke either a positive or a negative change in the targeted group of properties. One of the main desired reactions is, above all, cross-linking, especially when an improvement in the material’s characteristics is the primary objective [18,19]. The cross-linking process is underway when cross-linking prevails over degradation, i.e., the number of cross-linked chains is higher than the number of chain scissions. This observation was expanded upon by Gheysari et al. [23], who found that tested polymers demonstrated varying values of useful properties dependent upon the absorbed radiation dose (tensile strength rose with the increasing dose while elongation at break decreased). The actuator of these changes was the cross-linking process, which resulted in the creation of free radicals (breakup of C-H bonds) that subsequently recombined into a spatial network due to the linking of two free radicals together (C-C bond) with neighboring chains. As a result, mechanical properties were changed, which corresponds with the conclusions of other authors [18,24,25,26]. In the case of some polymers, an addition of a polyfunctional monomer is necessary to increase the initiation and recombination tendency of its radicals. For injection-molded parts, this additive is mixed into the polymer blend before the injection molding process [27]. This problem was studied by Malinowski [28], who reached the conclusion that exposing polybutylene adipate terephthalate (PBAT) filled by polyfunctional monomer triallyl isocyanurate (TAIC) to high-energy radiation led to cross-linking, which resulted in the creation of a significant gel fraction and, thus, the increase in tensile strength. Comparable results with regard to free radical recombination in polymers with polyfunctional monomers were observed in other studies as well [27,29].

Besides the cross-linking, irradiated polymers also face degradation processes, which can result in the worsening of useful properties. As shown in studies by Hama et al. [30] and other authors [31,32], one of these degradation processes is oxidation, which can cause the decline of the mechanical properties. On the other hand, degradation can also lead to the more frequent creation of carbonyl functional groups on the polymer surface [5,6], which can be useful for the improvement of adhesive properties and wettability of the surface. As can be seen, the potential of radiation cross-linking is quite high. Due to its universal nature, both the positive and the negative influences can be used in technical practice, one for the improvement of mechanical properties and the other for the modification of the surface layer. However, this universality is strongly dependent on the absorbed radiation dose, especially the ratio between both coexisting processes, i.e., cross-linking and degradation (oxidation). Up to now, many important studies have been written on the topic of the influence of high-energy radiation on the useful properties of polymer materials, e.g., studies of Holik, Danek, Manas et al. [33,34] and other authors [5,18,19,20,21,22,23,24,25,26,27,28,29]. Nevertheless, the aforementioned correlation between the absorbed radiation dose and the required surface and mechanical properties has not yet been comprehensively investigated.

Hence, the main goal of this study is to comprehensively describe the influence of radiation cross-linking (by suitably designed regression models) on the useful properties of injection-molded parts manufactured from commodity and technical plastics (PE and PA). Furthermore, it is necessary to investigate the universality of this method, especially regarding its ability to modify both the surface (free surface energy and adhesive properties) and the mechanical (tensile and bending strength) properties in a wide spectrum of working temperatures. The designed regression models could make finding the optimal value of applied radiation dose with regard to the required surface and mechanical properties of injection-molded parts easier.

## 2. Materials and Methods

### 2.1. Materials and Specimen Preparation

Verification of high-energy radiation’s influence on the useful properties of injection-molded parts was performed on one representative from group of commodity thermoplastics, specifically high-density polyethylene (HDPE) with trade name DOW HDPE 25055E provided by DOW (Midland, MI, USA). The other representative was chosen out of the technical plastics group, specifically, polyamide 66 (PA66) filled by 30 wt. % of glass fibers with trade name V-PTS-CREAMID-A3H7.2G6*M0129A provided by PTS (Adelshofen, Germany). The PA66 was also filled with polyfunctional monomer TAIC, which was performed to increase the recombination frequency of polymer radicals [27,28,29,34].

Specimens were molded using the Arburg Allrounder 420C and 470H (Loßburg, Germany). The cavities of the tools (injection molds) were machined with specific dimensions, which are given by the individual standards for testing mechanical and surface properties [35,36,37,38] and the strength of bonded joints in shear stressing [39]. The injection molding process parameters for each material were chosen according to the manufacturer’s recommendations and can be seen in Table 1.

### 2.2. Modification of Specimens by High-Energy Radiation

After the manufacturing, the specimens were exposed to high-energy radiation, specifically an electron beam produced by an electron accelerator (β radiation). The irradiation was performed in standard atmospheric conditions at ambient temperature in BGS Beta-Gamma Service, Saal an der Donau, Germany. Source of radiation was high-voltage electron accelerator, type Rhodotron 10 MeV–200 kW (Tongeren, Belgium). Magnitude and range of doses were set according to the industrial practice to 33, 66, 99, 132, 165, and 198 kGy. Since the thickness of modified samples was, at maximum, 10 mm, the one-sided irradiation method could be used (the penetration depth of β radiation was determined based on the density of irradiated material and energy of the electron beam) [3,36]. In order to prevent the induction of thermal stresses in the irradiated specimens, the exposure was performed in cycles (the specimens were exposed to 33 kGy each cycle). The magnitude of the radiation dose was measured by Nylon FTN 60-00 dosimeter (Goleta, CA, USA) [40]. The subsequent analysis of absorbed dose was performed by Genesys 5 spectrometer in accordance with ASTM 51261 [41].

### 2.3. Determination of Surface and Adhesive Properties

The influence of high-energy radiation on the wettability of injection-molded surfaces was determined and quantified by its free surface energy and the subsequent spectroscopic analysis that focused on the degree of oxidation. The influence of radiation on the adhesive properties of injection-molded surfaces was determined by change in the strength of bonded joints in shear and the type of failure.

#### 2.3.1. Free Surface Energy

The determination of free surface energy of the tested specimens was performed by regression model Owens–Wendt–Rabel–Kaelble (OWRK) [5,40,42,43,44,45,46], which uses the values of the wetting contact angles. The individual values of wetting contact angles were measured by sessile drop method on SeeSystem device made by Advex Instruments (Brno, Czech Republic). Each wetting contact angle measurement was performed according to CSN EN 15802 [38]. Three reference liquids with varying surface tension (distilled water: 72.8 mJ/m^2^, glycerine: 64 mJ/m^2^, ethylene glycol: 48 mJ/m^2^) were used [46]. Each specimen was measured 15 times for each reference liquid. Reference liquids’ drops were applied on surface layer by micropipette (volume of each applied drop was 4 µL).

**Figure 1:**
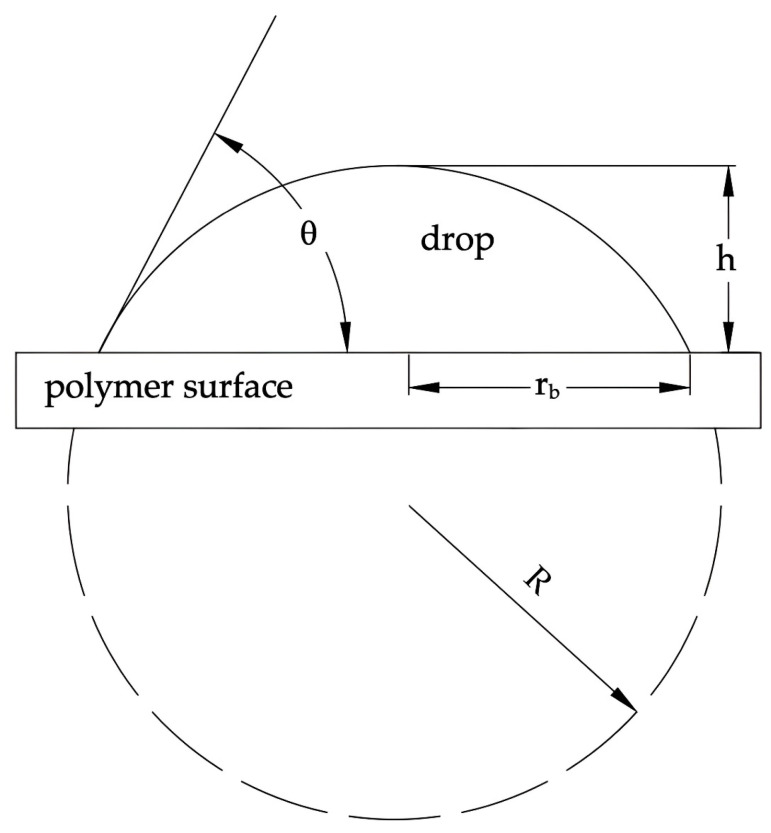
Droplet profile analysis: θ—wetting contact angle; h—droplet height, r_b_—droplet radius at contact point; and R—entire droplet radius [5,40].

The analysis of drops profile (Figure 1) was used in following equations, which led to determination of wetting contact angles [5,40,47]:

(1) $$h=R\left(1-\cos\theta \right),$$

(2) $$r_{b}=\mathrm{Rsin}\theta ,$$

and

(3) $$\frac{h}{r_{b}}=\frac{1-\cos\theta }{\sin\theta }=\tan\left(\frac{\theta }{2}\right).$$

#### 2.3.2. Fourier-Transform Infrared (FTIR) Spectroscopy

The degree of specimens’ surface layer changes was investigated by ATR-FTIR spectroscopy. This was performed on Nicolet iS50 FTIR device (Thermo Scientific^TM^, Waltham, MA, USA) [40] equipped with diamond ATR crystal. The spectra were recorded with 4 cm^−1^ resolution and 60 scans, while the evaluation was performed by Omnic^®^ software (version 9.3.32, Thermo Scientific^TM^, Waltham, MA, USA). Specifically, the spectra from three different places of the sample were acquired, and their baseline was corrected by means of automatic algorithm of the Omnics software. From these spectra, an average spectrum of the sample was calculated.

#### 2.3.3. Strength of Bonded Joints in Shear

**Figure 2:**
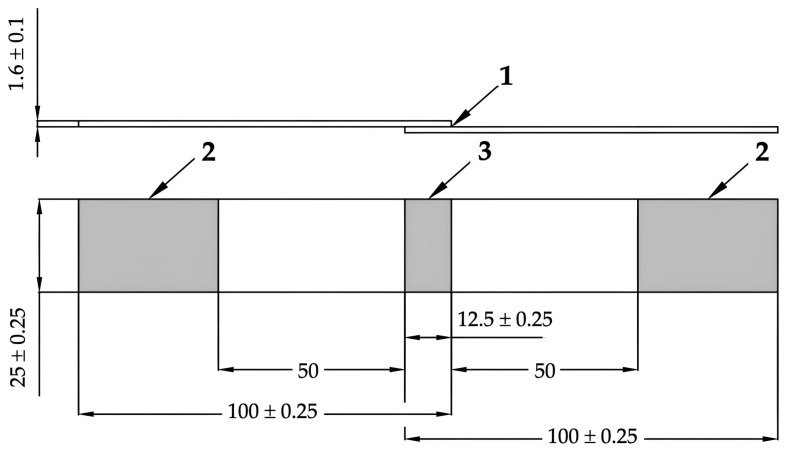
Bonded joint (dimensions in mm): (1) adhesive layer; (2) area of test machine grips; (3) shear area [39,40].

The creation of bonded joints, including shape and dimensions of specimens (Figure 2), was performed in accordance with CSN EN 1456 [39]. The specimens were bonded by commercially available adhesive with cyanoacrylate basis PR100 manufactured by 3M (Saint Paul, MN, USA). A consistent thickness of the adhesive layer was ensured by spacers placed between the bonded samples. The shear strength of joint’s bond was measured by testing on Zwick 1456 (ZwickRoell, Ulm, Germany) with crossbar velocity of 50 mm/min. The bonded joint was symmetrically placed in grips (distance between grips was (50 ± 1) mm). The spacers were used in order to ensure the point of force application was in bonded joint’s plane. The measured data were evaluated by TestXpert^®^ II software (version 2.1, ZwickRoell, Ulm, Germany).

#### 2.3.4. Analysis of Bonded Surfaces

The analysis of failure type of bonded joints was performed by evaluation of bonded joints’ images after strength testing by optical profilometer NewView 8000 (Zygo, Middlefield, OH, USA). The profilometer used interferometric scanning method and its vertical variation. The measurement is based on phase shift (use of white and monochromatic light). Speed of vertical scanning was 96 µm/s, and Streamlined Mx^TM^ software (version 8.0.0.33, Zygo, Middlefield, OH, USA) was used for processing of measured data.

### 2.4. Measurement of Mechanical Properties and Gel Content

The evaluation and quantification of the influence of high-energy radiation on the mechanical properties of tested specimens was performed by the investigation of tensile and bending strength, while the determination of cross-linked phase was performed by gel test. As injection-molded parts modified by high-energy radiation can be used in higher working temperatures, these properties were also tested under wide spectrum of working temperatures.

#### 2.4.1. Mechanical Properties

The testing of mechanical properties was performed by tensile strength and three-point bending test on universal testing machine Zwick 1456 (ZwickRoell, Ulm, Germany) in accordance with CSN EN ISO 527-1 [35], CSN EN 527-2 [36], and CSN EN ISO 178 [37] standards. The working temperatures for tensile strength and three-point bending tests can be seen in Table 2. A temperature chamber W91255 (ZwickRoell, Ulm, Germany) was used to heat the test specimens (in the case of tests at elevated temperatures). The test speed was set to 50 mm/min (tensile strength) and 5 mm/min (bending test). Distance between the supports for three-point bending test was (64 ± 1) mm. As with the testing of bonded joint strength, the measured data were evaluated by TestXpert^®^ II software (version 2.1, ZwickRoell, Ulm, Germany).

#### 2.4.2. Gel Content (Degree of Cross-Linking)

The determination of degree of cross-linking was performed by gel test in accordance with ASTM D2765 standard–Test Method C [48]. In this case, a 0.5 g of solid sample-cut from the whole modified specimen (measured to five decimal numbers) was mixed with 100 mL of solvent, i.e., xylene. The solvent dissolved amorphous part of tested polymers, while the cross-linked part remained undissolved. The mixture was boiled for 24 h. After that, the gel and the dissolved phase were separated. After separation, the gel (undissolved cross-linked part) was dried for 8 h in vacuum at 100 °C. Dried remnant of sample was once again weighted to five decimal numbers and compared with original weight. Degree of cross-linking was then determined from following equation [48,49]:

(4) $$G_{i}=\frac{m_{3}-m_{1}}{m_{2}-m_{1}}\cdot 100,$$

in which G_i_ represents the degree of cross-linking of tested sample (in percent); m_1_ is the weight of equipment (in milligrams); m_2_ is the overall weight of original sample and equipment (in milligrams), and m_3_ is the overall weight of sample’s remnant and equipment.

## 3. Results

The individual measurements were performed 15 times at atmospheric conditions and room temperature (23 °C). Based on measured data, a suitable regression model together with parameters was prepared to describe the influence of radiation dose on observed characteristics. The models were designed with the help of the following software: Minitab^®^17 (Minitab Inc., State College, PA, USA), QC-Expert 3.3 (TriloByte, Staré Hradiště, Czech Republic). The designed model has the following form:

(5) $$y=b_{0}+b_{1}x+b_{2}x^{2}$$

where y is the observed characteristic, x is the radiation dose (kGy), and b_0_, b_1_, b_2_ are the estimates of regression parameters.

During the search for a regression model, a test of statistical significance was performed. The output of regression model testing was the “rejection of hypothesis of insignificance”. Furthermore, a calculation of “predicted correlation coefficient” and “median quadratic error of prediction” was performed with the intent to estimate the regression parameters and find the type of regression function on confidential level 1 − α = 0.9, i.e., α = 0.1. This step, i.e., choice of α = 0.1, was necessary to correctly process the results, as due to the difficult preparation of specimens, a certain noise in the data must be assumed. At the end of data processing, a regression triplet was tested [50]. The subsequent spatial display of designed regression models was performed by OriginPro^®^ 2023 software (version 10.0, OriginLab, Northhampton, MA, USA).

### 3.1. Surface and Adhesive Properties

The effect of β radiation on the surface properties of tested materials was evaluated by the free surface energy since previously submitted studies [5,7,40] indicate that a high value of the free surface energy is the key factor for the good wettability of surfaces and the subsequent creation of quality adhesive bonds, which significantly affect the load capacity of bonded joints. For this reason, the load capacity of bonded joints was chosen as demonstrative test of practical application of β radiation effect on the adhesive properties.

**Figure 3:**
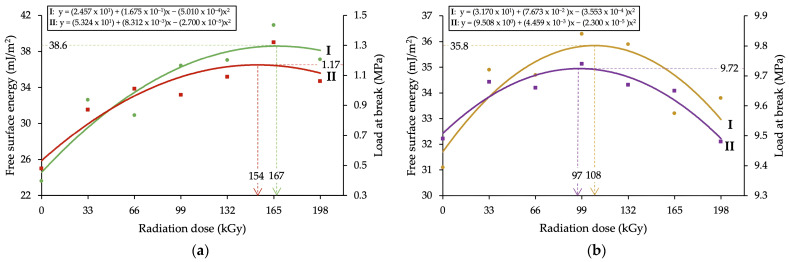
Proposed regression models–the effect of radiation dose on surface and adhesion properties: (**aI**) HDPE, free surface energy; (**aII**) HDPE, load-bearing of adhered joints; (**bI**) PA66, free surface energy; (**bII**) PA66, load-bearing of adhered joints.

The models’ designed parameters for the description of the change in free surface energy and strength of the bonded join in dependence on absorbed radiation dose can be seen in Table 3, while individual models are displayed in Figure 3. The characteristics of designed regression models are shown in Table 4.

The results shown in Table 3 and Figure 3 demonstrate that β radiation influences the value of free surface energy. Out of both tested polymers, a higher growth of free surface energy was found in HDPE, in which the value rose from 24.6 mJ/m^2^ to 38.6 mJ/m^2^, which was a total increase of 57%. In the case of PA66, the growth was 13%, which was not as impressive. The increase in free surface energy influenced adhesive properties of tested polymers, which resulted in an increase in the shear strength in bonded joints.

The shear strength of HDPE modified by radiation rose from 0.53 MPa to 1.17 MPa, which was an increase of 120%. For PA66, the growth of shear strength was significantly lower, approximately 3%. The highest growth for both polymers and each observed characteristic was found in materials irradiated by higher doses of radiation, specifically the doses higher than 132 kGy for HDPE and the doses in the range of 99 to 132 kGy for PA66.

Table 4 shows the characteristics of designed regression models designated for the description of changes in the surface and adhesive properties of tested polymers modified by the β radiation. As can be interpreted from the results, the designed models for the description of β radiation effect on the magnitude of free surface energy and the strength of bonded joints are significant and correct. The residues demonstrate homoskedasticity, a normal distribution, and insignificant autocorrelation.

**Figure 4:**
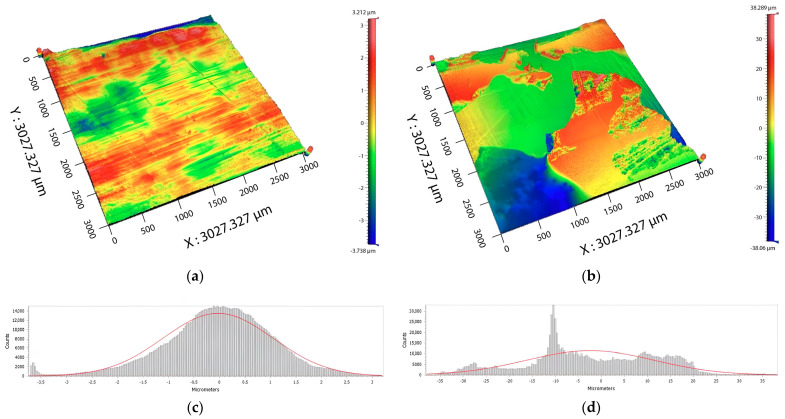
The bonded surface for HDPE after shear strength test: (**a**) Image of original surface; (**b**) image of surface modified by β radiation; (**c**) topography of non-modified surface; (**d**) topography of surface modified by β radiation.

**Figure 5:**
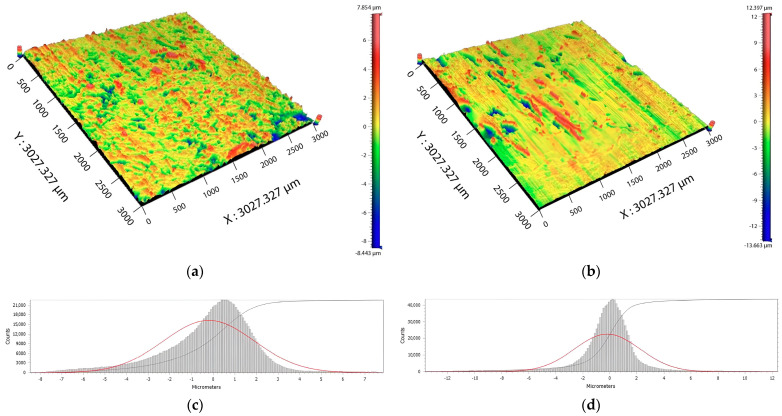
The bonded surface for PA66 after shear strength test: (**a**) Image of original surface; (**b**) image of surface modified by β radiation; (**c**) topography of non-modified surface; (**d**) topography of surface modified by β radiation.

Besides the growth of the strength of bonded joints and adhesive properties, the effect of the radiation modification can also be observed in the change in failure type in the bonded joint. Figure 4 and Figure 5 show images of bonded surfaces after the strength test. For HDPE, the non-modified surfaces generally experienced failure on the phase interface of adherent/adhesive (Figure 4a). Due to irradiation, adhesive properties were improved, which also impacted the change in failure type. After the modification by β radiation, a mixed failure type was observed in HDPE, i.e., a combination of adhesive and cohesive failure (Figure 4b). Figure 4b displays the bonded surface (after shear strength testing), which was modified by β radiation. The blue and red areas indicate assumed adhesive failure, while green and yellow areas indicate cohesive failure in a layer of adhesive (height of adhesive was 80 µm). In the case of PA66, it is difficult to unequivocally determine the type of failure (Figure 5a,b). However, due to irradiation, the change in the topography of the bonded surface occurred in both HDPE (Figure 4c,d) and PA66 (Figure 5c,d).

As can be seen in Figure 4a,c, the non-modified surface has a distribution of Z coordinates quite close to the standard normal distribution with parameters µ = 0 and σ = 1. This is given not only by the normality test, according to Anderson–Darling, which has not refused normality, but also from the shape of the histogram for the Z coordinate. On the other hand, Figure 4b,d shows the surface of the specimen modified by the β radiation, which shows a significant breach of normality of the Z coordinate. The Anderson–Darling test showed that the normality of the Z coordinate was refused. This can also be interpreted from the asymmetrical shape of a given histogram.

As can be seen in Figure 5a,c, the non-modified surface demonstrates the Z coordinate distribution that is very close to the standard normal distribution with parameters µ = 0 and σ = 1. The coordinate was once again tested by the Anderson–Darling test, which has not refused normality. However, it is also possible to say that the Z coordinate was slightly sloped. This observation does not appear significant. The surface in Figure 5b,d, which was modified by β radiation, does not demonstrate a breach of normality of the Z coordinate. The repeated application of the Anderson–Darling test did not lead to the refusal of the Z coordinate.

**Figure 6:**
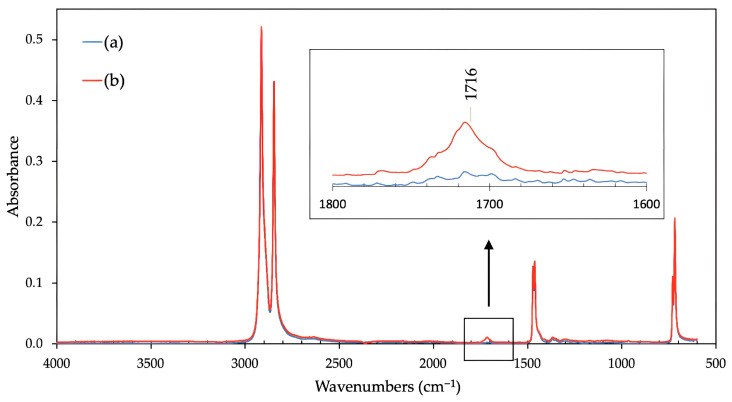
The infrared spectra: (a) HDPE, untreated; (b) HDPE, after β radiation treatment.

**Figure 7:**
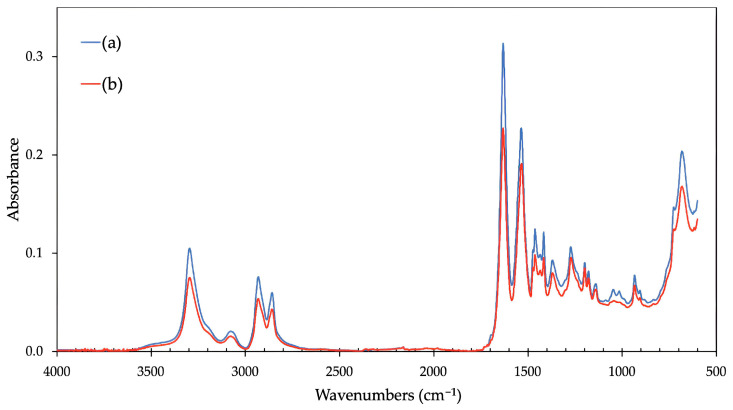
The infrared spectra: (a) PA66, untreated; (b) PA66, after βradiation treatment.

Figure 6 and Figure 7 show the results from infrared spectroscopy. The evaluation of spectra of the non-modified HDPE (Figure 6a) and the material modified by the β radiation demonstrated the characteristic absorption bands in the range of 1680 cm^−1^ to 1740 cm^−1^, which confirms the expected creation of functional carbonyl groups in tested material (Figure 6b). These results correspond with an earlier study [5], which focused on the change in surface layer properties, including the oxidation and relative representation of carbonyl and hydroxyl functional groups, dependent upon the absorbed dose of high-energy radiation.

In the case of PA66 modified by the β radiation, there are some notable changes between the spectra of the untreated and irradiated samples (Figure 7). Specifically, a decreased intensity of the peak area at 1684 cm^−1^, lower ratio of Amide I (1632 cm^−1^) to Amide II (1537 cm^−1^) respective bands, and changes in the absorption bands between 1060 and 1000 cm^−1^ can be observed in the spectrum of the irradiated sample (Figure 7b). This indicates the reduction of C=O groups in the polymer chain and possible conformational changes.

### 3.2. Mechanical Properties

The changes in mechanical properties induced by the β radiation were determined by tensile and bending strength tests. Both tested characteristics were measured in a wide spectrum of working temperatures (see Table 2). The designed parameters of the regression model designated for the description of changes in tensile and bending strength dependent upon the absorbed dose can be seen in Table 5 and Table 6.

**Figure 8:**
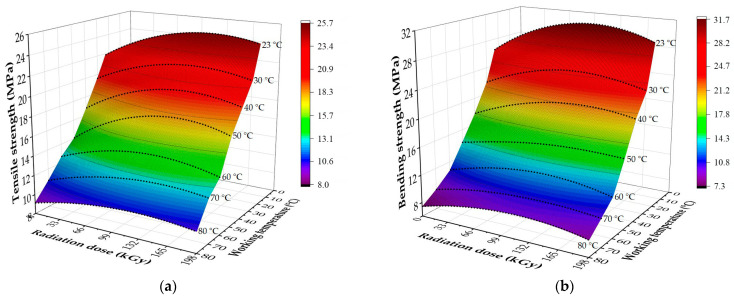

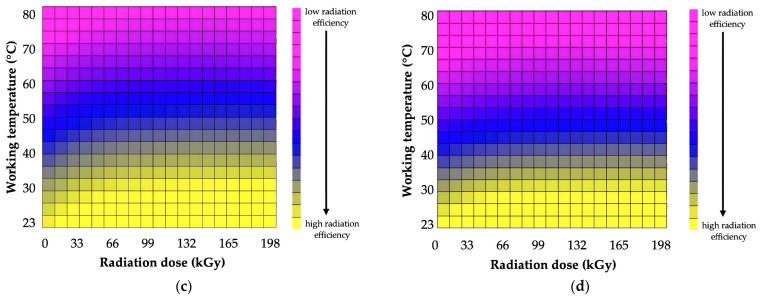
Mechanical properties of HDPE: (**a**) spatial portrayal of regression models (tensile strength); (**b**) spatial description of regression models (bending strength); (**c**) responsive area (tensile strength); (**d**) responsive area (bending strength).

**Figure 9:**
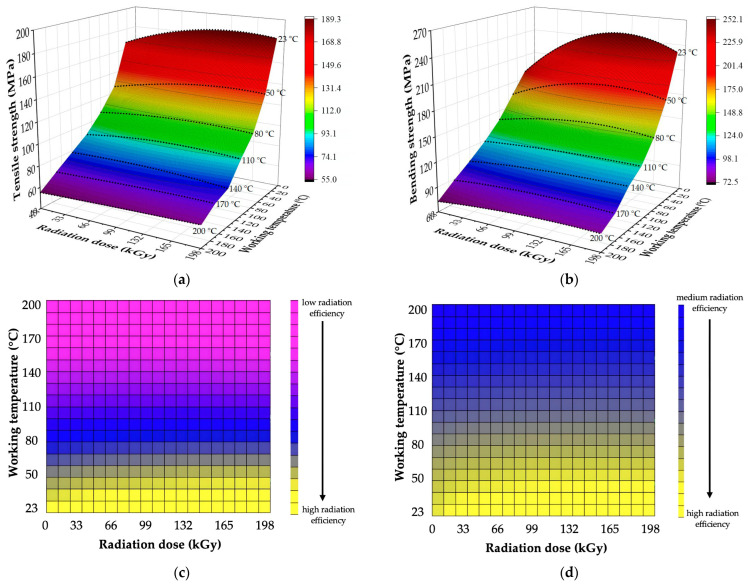
Mechanical properties of PA66: (**a**) spatial portrayal of regression models (tensile strength); (**b**) spatial description of regression models (bending strength); (**c**) responsive area (tensile strength); (**d**) responsive area (bending strength).

**Figure 10:**
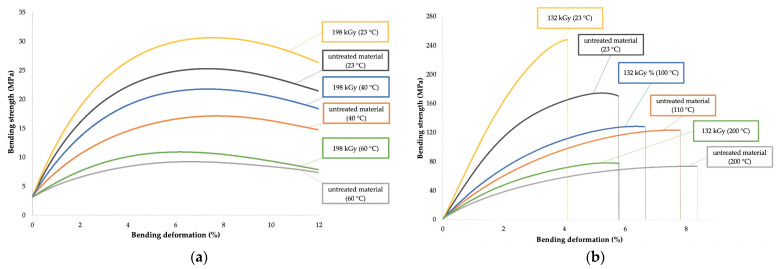
The dependence of bending stress on deformation: (**a**) HDPE; (**b**) PA66.

A spatial portrayal of designed regression models with their respective areas, which characterize the degree of influence of radiation on given characteristics (high, medium, and low influence), can be seen in Figure 8 and Figure 9, while examples of bending curves are presented in Figure 10. The characteristics of designed regression models are shown in Table 7 and Table 8.

The results displayed in Table 5, Figure 8 and Figure 10 show that β radiation increased the tensile and bending strength in HDPE for a wide spectrum of working temperatures. In the case of the tensile strength, the increase was in the range of 13 to 28%, depending on the applied radiation dose and working temperature (Table 5 and Figure 8a). For example, at ambient temperature, the tensile strength grew by 3.5 MPa, while only a 1.4 MPa increase was measured for tests conducted at 80 °C. Regarding the bending strength, the growth was in the range of 15 to 27%, dependent on the radiation dose and the working temperature (Table 5, Figure 8b and Figure 10a). For example, at ambient temperature, the bending strength grew by 5.2 MPa, while at the highest working temperature, the growth was only 2.1 MPa. On responsive areas (Figure 8c,d), the effect of radiation on the tensile and bending strength can be observed for concrete working temperatures. Both tested characteristics revealed a medium-to-high effect of radiation for working temperatures up to 60 °C (yellow and blue area). The effect of radiation continually decreased for working temperatures over 60 °C (pink area).

The effect of β radiation on the tensile and bending strength of PA66 can be seen in Table 6 and Figure 9 and Figure 10. In the case of tensile strength, the irradiation led to an increase of up to 13%, dependent on the radiation dose and working temperature (Table 6 and Figure 9a). At ambient temperature, the tensile strength grew by 21.6 MPa, while at the highest temperature, the same characteristic rose only by 4.8 MPa. The increase in bending strength was up to 33%, dependent on the radiation dose and working temperature (Table 6, Figure 9b and Figure 10b). At the lowest tested temperature, the growth induced by radiation was 62.7 MPa, while at the highest tested temperature, it was 8.8 MPa. The responsive area (Figure 9c) displays that for tensile strength, the effect of radiation was medium or high for working temperatures up to 130 °C (yellow and blue area). For working temperatures over 130 °C, the effect of irradiation continually decreased (pink area). In the case of the bending strength (Figure 9d), the effect of radiation was medium or high up to the highest working temperature, i.e., 200 °C (yellow and blue area).

The characteristics of designed regression models for the description of changes in the mechanical properties of tested polymers induced by the β radiation can be seen in Table 7 and Table 8. The interpretation of measured results shows that the designed models for the description of β radiation’s influence on the magnitude of tensile and bending strength are significant and correct. The residues demonstrate homoskedasticity and normal distribution, and the autocorrelation is insignificant.

## 4. Discussion

This study is primarily focused on a quantitative description of changes in useful properties in injection-molded parts due to irradiation by β radiation. Specimens were manufactured out of one representative for the commodity plastics (HDPE) and one representative for the technical plastics (PA66). First, the properties of the surface layer, such as free surface energy and adhesion, were tested. The measured results indicate that the β radiation influences the properties of the surface layer (Table 3 and Figure 3). In the case of free surface energy in HDPE, the highest growth was seen in specimens irradiated by higher doses of radiation (more than 132 kGy) (Table 3 and Figure 3a). On the other hand, for PA66, the highest growth was observed in specimens irradiated by the medium intensity of radiation, i.e., 99 to 132 kGy (Table 3 and Figure 3b). The increase in free surface energy (in comparison with the non-modified material) was up to 57% for HDPE and up to 13% for PA66. The change in free surface energy value had a significant effect on the adhesive properties of tested materials. The practical effect of β radiation on adhesive properties was tested by evaluation of the shear strength of bonded joints. Figure 3 demonstrates that the β radiation modification influenced the shear strength of bonded joints for both HDPE and PA66. In specific cases, the strength of the bonded joint rose by 120% (Table 3 and Figure 3).

The presented changes in terms of free surface energy and adhesive properties taken in the context of the strength of bonded joints were most likely caused by oxidation, which could have occurred during the irradiation process or after it. Oxidation is one of many secondary reactions which can occur when β radiation interacts with polymers. A significant factor of this study is that the process of specimen modification was the same as with common industrial applications performed at ambient temperature and in standard atmospheric conditions (with oxygen). In this case, the free radicals created due to irradiation could easily react with oxygen molecules, which results in the creation of peroxide radicals that could lead to oxidation. Moreover, it is the oxygen and humidity which help oxidation reactions (oxidation, oxidative scission). As stated by Rivaton et al. [51], most post-radiation effects are driven by the migration of radicals from crystalline areas to amorphous/crystalline mesophases, where the radicals remain more accessible for oxygen. As mentioned previously, oxidation can occur during the irradiation process itself (contemporaneously with cross-linking and scission) or after it if the polymer can react with oxygen. This conclusion is supported by the results of infrared spectra (Figure 6) for HDPE and corresponds with conclusions reached by Hama et al. [30], Carpentieri et al. [52], and Costa et al. [53].

The oxidation usually occurs together with oxidative degradation, and the combination of these effects can cause a different color (yellow) and fragility (or decrease in other mechanical properties) in the materials; thus, it is best to avoid these interactions [20,21]. However, new functional groups (carbonyl and others) can enhance the surface with new properties which can find their application in practice. Among these properties are adhesion, the increase in polarity, and others, which all positively affect the load capability of bonded joints (Figure 3). The change in adhesive properties is shown in Figure 4 and Figure 5, which both display bonded surfaces after shear strength testing. The recorded changes were most significant in HDPE (Figure 4), in which the non-modified surface displayed mostly adhesive failure, i.e., the failure occurred at the adhesive/adherent interface (Figure 4a). After the modification by β radiation, the failure changed to combined, i.e., a combination of adhesive and cohesive failure (Figure 4b). Besides the change in the type of failure, the irradiation also led to a change in the topography of bonded surfaces (Figure 4c,d). The change in the type of bond failure corresponds with improved adhesive bonds. In the case of PA66 (Figure 5a,b), the type of failure was not unequivocally evident. On the other hand, irradiation definitely led to changes in the topography of the bonded surface (Figure 5c,d).

**Figure 11:**
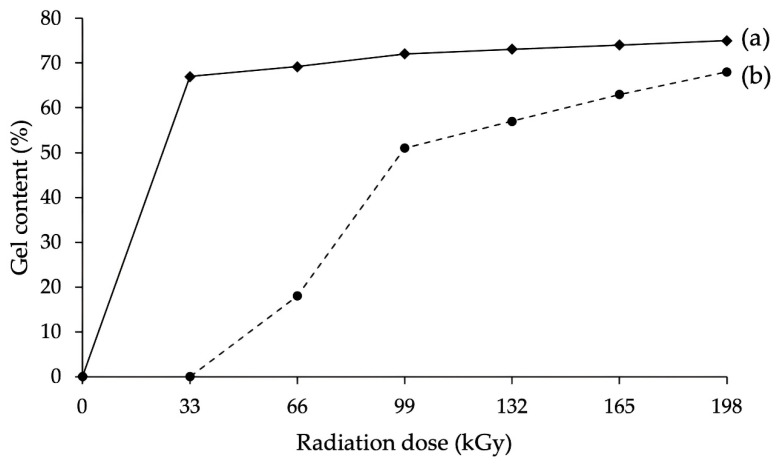
The effect of radiation on gel content: (a) PA66; (b) HDPE.

The second tested group focused on mechanical properties, such as tensile and bending strength. Measured results indicate that β radiation influences even the mechanical properties in a wide spectrum of working temperatures (Table 5 and Table 6, Figure 8, Figure 9 and Figure 10). In both cases, the characteristics increased due to irradiation for both tested materials. For tensile strength, the growth was almost 28% (Figure 8a and Figure 9a) in comparison with non-altered material. For bending strength, this increase was even greater, up to 33% (Figure 8b, Figure 9b and Figure 10). The changes in mechanical properties induced by the β radiation correspond with the content of the cross-linked phase (gel), which also rose with increasing radiation dose (Figure 11). Furthermore, these results correspond with the findings of other authors [18,33,34,54,55], who focused on the effects of high-energy radiation on the mechanical properties of polymers. The increasing content of gel induced by radiation and its influence on mechanical properties was also detected by Lee et al. [22], who recorded an increase in tensile strength together with the rising content of gel, which was caused by radiation. The aforementioned changes (improvements) of mechanical properties were caused above all by cross-linking, which is one of the secondary processes that occur in polymer materials (prevalently in amorphous areas [56]) due to β irradiation [33,34,54,55,57].

When the tested polymers were exposed to radiation, C-H bonds started to scission (release of the hydrogen atom), which led to the creation of free radicals. Afterward, gradual bonding (creation of C-C bond) of two free radicals of neighboring chains commenced. In the end, a 3D spatial network was created in which the polymer chains were interconnected. This spatial network was the bearer of improvements in the mechanical properties of tested polymers. The creation of a spatial network is proved by the aforementioned results of gel content (insoluble phase) increase due to radiation (Figure 11). In the case of PA66 with 30 wt. % of glass fibers, the irradiation incurred improvement of adhesion between individual fibers, which resulted in the increase in mechanical properties [18,34,55].

The description of changes in properties of tested materials due to irradiation was performed by suitably designed regression models. All regression models were tested with regression triplet. The regression models were significant and correct; residues demonstrated homoskedasticity and normal distribution, and autocorrelation was insignificant. The designed regression models showed that the highest growth of the given parameter was reached in specimens exposed to radiation dose lower than 198 kGy, i.e., after the maximum was reached, the observed parameter decreased with increasing radiation dose (Figure 3, Figure 8a,b and Figure 9a,b). This course was also noted in studies of other authors [58], who used the second-degree polynomial equation (quadratic polynomial) to describe the changes in properties of specimens exposed to high-energy radiation. This effect can be explained by parameter G, which is commonly used in practice to determine the reactions ongoing in the material during the irradiation. As presented by Makuuchi, Cheng [20], and Drobny [21], parameter G can be defined as the chemical gain of radiation in dependence on the number of reacting molecules per 100 eV of absorbed energy. At a certain point, the regression curve (extreme of function) experiences a breakpoint, following which the degradation reactions start to prevail over cross-linking, which results in a decline of useful properties and an overall decline in the regression curve. In the case of free surface energy and adhesive properties (Figure 3), a gradual increase in radiation dose led to a point where chain scission started to prevail over cross-linking. At this point, even the chains that cross-linked started to undergo scission, and it resulted in a decrease in free surface energy and an increase in the hydrophobic nature of the surface. This corresponds with findings presented by Egghe et al. [59]. This is also true for mechanical properties which started to decrease with the increasing radiation dose after reaching a certain breakpoint (specific radiation dose) (Figure 8 and Figure 9) despite the continuous growth of gel content. This effect was recorded in studies of Gheysari and Behjat [23,60]. Although the exposure to higher radiation doses led to a gradual increase in the cross-linking phase, the degradation processes started to prevail at a certain point. This likely caused the decrease in the quality of the created spatial network and, thus, the decline of useful properties.

**Figure 12:**
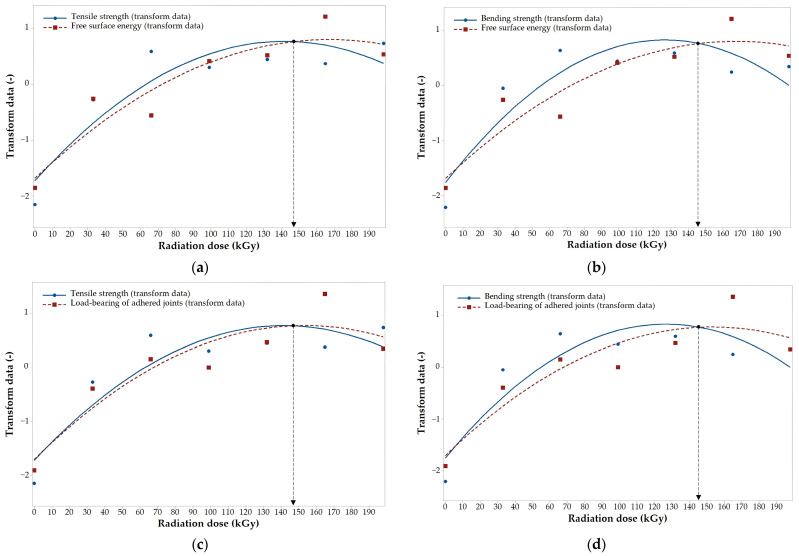
The optimal dose for HDPE: (**a**) tensile strength/free surface energy; (**b**) bending strength/free surface energy; (**c**) tensile strength/load-bearing of adhered joints; (**d**) bending strength/load-bearing of adhered joints.

**Figure 13:**
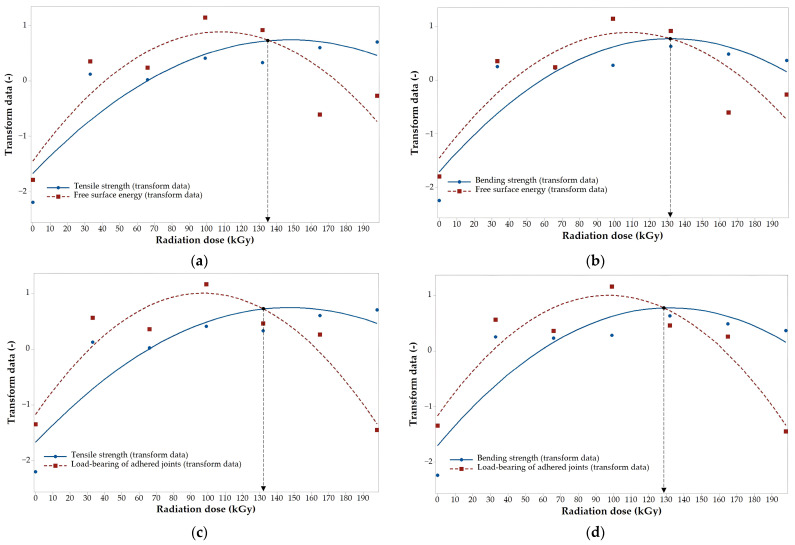
The optimal dose for PA66: (**a**) tensile strength/free surface energy; (**b**) bending strength/free surface energy; (**c**) tensile strength/load-bearing of adhered joints; (**d**) bending strength/load-bearing of adhered joints.

The designed regression models can be used to find a suitable (optimal) dose of radiation, which, when applied, leads to the best results in both the surface properties and the mechanical properties (Figure 12 and Figure 13). The optimal dose was determined by standard parameters (by transformed data), which were calculated from specific values subtracted by the average value and then divided by standard deviation. Figure 12 and Figure 13 show optimal values which were close to the extremes of functions. In the case of HDPE, the optimal dose was in the range of 145 to 150 kGy, depending on the combination of surface and mechanical properties (Figure 12). The optimal dose for PA66 was found in the range of 128 to 135 kGy, depending on the combination of surface and mechanical properties (Figure 13).

## 5. Conclusions

Tests and measurements performed in this study lead to the following conclusions:

- The modification of polymer materials by β radiation leads to the improvement of useful properties of injection-molded parts.
- Well-chosen doses of radiation can lead to improvement of both the mechanical and the surface properties.
- The designed regression models can be used as a suitable tool for choosing the optimal dose of radiation in terms of the required properties of the given part and its application in a specific working environment.

Future research in this area should focus on the effects of very low doses of radiation (in the range of 0 to 20 kGy) on the useful properties of injection-molded parts and the stability of gained properties, which is specifically true for surface layer properties.

## Figures and Tables

**Table 1:** The injection molding process parameters.

Processing Conditions	HDPE	PA66
Injection Rate (mm/s)	60	60
Injection Pressure (MPa)	80	88
Holding Pressure (MPa)	60	70
Holding Time (s)	30	25
Cooling Time (s)	20	30
Mold Temperature (°C)	40	75
**Plastic Unit Temperature Bands**		
Zone 1 (°C)	200	265
Zone 2 (°C)	205	280
Zone 3 (°C)	210	285
Zone 4 (°C)	225	290

**Table 2:** The mechanical properties–working temperatures.

Material	Mechanical Properties (MPa)	Working Temperatures (°C)
HDPE	Tensile and Bending Strength	23, 30, 40, 50, 60, 70, 80
PA66		23, 50, 80, 110, 140, 170, 200

**Table 3:** The estimations of regression parameters–surface and adhesive properties.

Material	Tested Parameter	Estimations of Regression Parameters		
		b_0_	b_1_	b_2_
HDPE	Free Surface Energy (mJ/m^2^)	2.457 × 10^1^	1.675 × 10^−1^	−5.010 × 10^−4^
	Load-Bearing of Adhered Joints (MPa)	5.324 × 10^−1^	8.312 × 10^−3^	−2.700 × 10^−5^
PA66	Free Surface Energy (mJ/m^2^)	3.170 × 10^1^	7.673 × 10^−2^	−3.553 × 10^−4^
	Load-Bearing of Adhered Joints (MPa)	9.508 × 10^0^	4.459 × 10^−3^	−2.300 × 10^−5^

**Table 4:** The characteristics of designed regression models–surface and adhesive properties.

Parameters	HDPE		PA66	
	Free Surface Energy	Load-Bearing of Adhered Joints	Free Surface Energy	Load-Bearing of Adhered Joints
Coefficient of Multiple Correlation	9.352 × 10^−1^	9.223 × 10^−1^	8.579 × 10^−1^	9.424 × 10^−1^
Coefficient of Determination	8.745 × 10^−1^	8.507 × 10^−1^	7.360 × 10^−1^	8.881 × 10^−1^
Predicted Correlation Coefficient	3.503 × 10^−1^	2.188 × 10^−1^	1.626 × 10^−1^	4.422 × 10^−1^
Mean Squared Error of Prediction	1.117 × 10^1^	3.016 × 10^−2^	3.763 × 10^0^	2.842 × 10^−3^
**Testing of Regression Triplet**				
Fisher–Snedecor Test of Model Significance	model is significant			
Scott’s Criteria of Multicollinearity	model is correct			
Cook–Weisberg Score Test for Heteroskedasticity	residue demonstrating homoskedasticity			
Jarque–Berra Test of Normality	residue has normal distribution			
Wald Test of Auto Correlation	autocorrelation is insignificant			
Durbin–Watson Test of Auto Correlation	negative autocorrelation of residues not demonstrated			

**Table 5:** The estimation of regression parameters–mechanical properties (HDPE).

Tested Property (MPa)	Working Temperature (°C)	Estimations of Regression Parameters		
		b_0_	b_1_	b_2_
Tensile Strength	23	2.189 × 10^1^	4.892 × 10^−2^	−1.727 × 10^−4^
	30	1.965 × 10^1^	3.950 × 10^−2^	−1.520 × 10^−4^
	40	1.674 × 10^1^	5.195 × 10^−2^	−1.968 × 10^−4^
	50	1.406 × 10^1^	5.952 × 10^−2^	−2.230 × 10^−4^
	60	1.267 × 10^1^	3.409 × 10^−2^	−1.432 × 10^−4^
	70	1.082 × 10^1^	2.835 × 10^−2^	−1.006 × 10^−4^
	80	9.150 × 10^0^	2.165 × 10^−2^	−8.527 × 10^−5^
Bending Strength	23	2.642 × 10^1^	8.323 × 10^−2^	−3.290 × 10^−4^
	30	2.183 × 10^1^	5.530 × 10^−2^	−2.285 × 10^−4^
	40	1.769 × 10^1^	5.519 × 10^−2^	−2.033 × 10^−4^
	50	1.410 × 10^1^	2.781 × 10^−2^	−9.073 × 10^−5^
	60	1.071 × 10^1^	4.004 × 10^−2^	−1.749 × 10^−4^
	70	8.898 × 10^0^	2.597 × 10^−2^	−1.028 × 10^−4^
	80	7.386 × 10^0^	3.517 × 10^−2^	−1.541 × 10^−4^

**Table 6:** The estimation of regression parameters–mechanical properties (PA66).

Tested Property (MPa)	Working Temperature (°C)	Estimations of Regression Parameters		
		b_0_	b_1_	b_2_
Tensile strength	23	1.669 × 10^2^	2.908 × 10^−1^	−9.806 × 10^−4^
	50	1.262 × 10^2^	1.735 × 10^−1^	−5.302 × 10^−4^
	80	1.056 × 10^2^	8.377 × 10^−2^	−3.280 × 10^−4^
	110	8.970 × 10^1^	9.665 × 10^−2^	−3.903 × 10^−4^
	140	7.853 × 10^1^	2.489 × 10^−2^	−2.274 × 10^−4^
	170	6.714 × 10^1^	2.771 × 10^−2^	−7.871 × 10^−5^
	200	5.525 × 10^1^	2.435 × 10^−2^	−7.980 × 10^−5^
Bending strength	23	1.889 × 10^2^	9.491 × 10^−1^	−3.594 × 10^−3^
	50	1.651 × 10^2^	4.370 × 10^−1^	−1.675 × 10^−3^
	80	1.385 × 10^2^	2.314 × 10^−1^	−9.030 × 10^−4^
	110	1.192 × 10^2^	6.753 × 10^−2^	−1.968 × 10^−4^
	140	1.032 × 10^2^	7.727 × 10^−2^	−1.793 × 10^−4^
	170	8.870 × 10^1^	5.942 × 10^−2^	−2.241 × 10^−4^
	200	7.257 × 10^1^	1.054 × 10^−1^	−3.170 × 10^−4^

**Table 7:** The characteristics of designed regression models (HDPE).

Parameters		Working Temperature (°C)						
		23	30	40	50	60	70	80
Coefficient of Multiple Correlation	Tensile strength	9.099 × 10^−1^	9.187 × 10^−1^	8.646 × 10^−1^	8.810 × 10^−1^	8.590 × 10^−1^	9.297 × 10^−1^	8.524 × 10^−1^
	Bending strength	9.121 × 10^−1^	9.266 × 10^−1^	8.771 × 10^−1^	9.445 × 10^−1^	8.315 × 10^−1^	8.631 × 10^−1^	8.756 × 10^−1^
Coefficient of Determination	Tensile strength	8.279 × 10^−1^	8.439 × 10^−1^	7.475 × 10^−1^	7.762 × 10^−1^	7.378 × 10^−1^	8.643 × 10^−1^	7.265 × 10^−1^
	Bending strength	8.318 × 10^−1^	8.586 × 10^−1^	7.693 × 10^−1^	8.922 × 10^−1^	6.913 × 10^−1^	7.449 × 10^−1^	7.666 × 10^−1^
Predicted Correlation Coefficient	Tensile strength	2.090 × 10^−2^	4.271 × 10^−3^	3.806 × 10^−1^	7.883 × 10^−2^	2.528 × 10^−1^	1.113 × 10^−1^	7.581 × 10^−1^
	Bending strength	3.031 × 10^−2^	1.986 × 10^−2^	2.075 × 10^−2^	4.968 × 10^−2^	6.614 × 10^−1^	3.936 × 10^−1^	2.980 × 10^−2^
Mean Squared Error of Prediction	Tensile strength	1.892 × 10^0^	9.023 × 10^−1^	2.781 × 10^0^	2.865 × 10^0^	8.863 × 10^−1^	3.503 × 10^−1^	5.207 × 10^−1^
	Bending strength	4.183 × 10^0^	1.185 × 10^0^	2.320 × 10^0^	4.701 × 10^−1^	1.476 × 10^0^	6.297 × 10^−1^	4.667 × 10^−1^
**Testing of Regression Triplet**								
Fisher–Snedecor Test of Model Significance		model is significant						
Scott’s Criteria of Multicollinearity		model is correct						
Cook–Weisberg Score Test for Heteroskedasticity		residue demonstrating homoskedasticity						
Jarque–Berra Test of Normality		residue has normal distribution						
Wald Test of Auto Correlation		autocorrelation is insignificant						
Durbin–Watson Test of Auto Correlation		negative autocorrelation of residues not demonstrated						

**Table 8:** The characteristics of designed regression models (PA66).

Parameters		Working Temperature (°C)						
		23	50	80	110	140	170	200
Coefficient of Multiple Correlation	Tensile strength	9.053 × 10^−1^	9.478 × 10^−1^	9.311 × 10^−1^	8.935 × 10^−1^	9.772 × 10^−1^	9.721 × 10^−1^	9.468 × 10^−1^
	Bending strength	8.888 × 10^−1^	8.509 × 10^−1^	8.679 × 10^−1^	9.759 × 10^−1^	9.801 × 10^−1^	8.549 × 10^−1^	9.559 × 10^−1^
Coefficient of Determination	Tensile strength	8.196 × 10^−1^	8.983 × 10^−1^	8.670 × 10^−1^	7.984 × 10^−1^	9.548 × 10^−1^	9.449 × 10^−1^	8.964 × 10^−1^
	Bending strength	7.900 × 10^−1^	7.240 × 10^−1^	7.532 × 10^−1^	9.523 × 10^−1^	9.605 × 10^−1^	7.308 × 10^−1^	9.138 × 10^−1^
Predicted Correlation Coefficient	Tensile strength	2.192 × 10^−2^	3.816 × 10^−1^	1.299 × 10^−2^	3.082 × 10^−2^	6.202 × 10^−1^	5.790 × 10^−1^	3.429 × 10^−1^
	Bending strength	7.791 × 10^−2^	9.397 × 10^−2^	2.320 × 10^−1^	7.983 × 10^−1^	7.307 × 10^−1^	1.908 × 10^−2^	1.739 × 10^−1^
Mean Squared Error of Prediction	Tensile strength	7.616 × 10^1^	1.040 × 10^1^	3.143 × 10^0^	5.599 × 10^0^	5.565 × 10^−1^	1.842 × 10^−1^	1.891 × 10^−1^
	Bending strength	6.953 × 10^2^	1.594 × 10^2^	4.653 × 10^1^	4.594 × 10^−1^	1.218 × 10^0^	2.006 × 10^0^	5.965 × 10^0^
**Testing of regression triplet**								
Fisher–Snedecor Test of Model Significance		model is significant						
Scott’s Criteria of Multicollinearity		model is correct						
Cook–Weisberg Score Test for Heteroskedasticity		residue demonstrating homoskedasticity						
Jarque–Berra Test of Normality		residue has normal distribution						
Wald Test of Auto Correlation		autocorrelation is insignificant						
Durbin–Watson Test of Auto Correlation		negative autocorrelation of residues not demonstrated						

## Data Availability

The data presented in this study are available upon request from the corresponding author.
